# Supplementary material for: Exploring RAB11A Pathway to Hinder Chronic Myeloid Leukemia-Induced Angiogenesis In Vivo
Source: Pharmaceutics. 2023 Feb 23;15(3):742. doi: 10.3390/pharmaceutics15030742 (PMC10056245; doi:10.3390/pharmaceutics15030742)
Supplement: Supplementary file 1 [file pharmaceutics-15-00742-s001.zip › pharmaceutics-2202853-supplementary.pdf]

Supplementary Materials

Exploring *RAB11A* Pathway to Hinder Chronic Myeloid Leukemia-Induced Angiogenesis In Vivo

Catarina Roma-Rodrigues [1,2,*], Alexandra R. Fernandes [1,2,*] and Pedro V. Baptista [1,2]

1 Associate Laboratory i4HB—Institute for Health and Bioeconomy, NOVA School of Science and Technology, NOVA University Lisbon, 2829-516 Caparica, Portugal
2 UCIBIO—Applied Molecular Biosciences Unit, Department of Life Sciences, NOVA School of Science and Technology, NOVA University Lisbon, 2829-516 Caparica, Portugal
* Correspondence: catromar@fct.unl.pt (C.R.-R.); ma.fernandes@fct.unl.pt (A.R.F.); Tel.: +351-212948530 (C.R.-R. & A.R.F.).

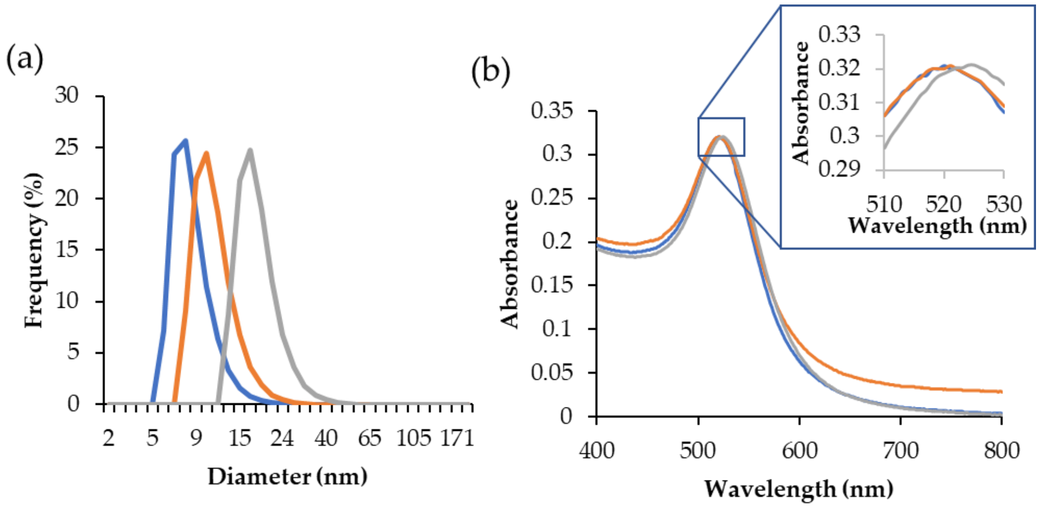

Figure S1. Characterization of gold nanoparticles (AuNPs). (**a**) Dynamic light scattering profile of the diameter of citrate-caped AuNPs (blue line), AuNPs covered with polyethylene glycol (AuNP@PEG, orange line), and AuNP@PEG functionalized with an shDNA anti-*RAB11A* (AuNP@RAB11A, grey line); (**b**) UV-Vis spectra of citrate caped AuNPs (blue line), AuNP@PEG (orange line), and AuNP@RAB11A (grey line).

An increased diameter of the AuNPs, with citrate caped AuNPs with an average diameter of 17.8 ± 0.1 nm, AuNP@PEG with an average diameter of 22.0 ± 0.4 nm and AuNP@RAB11A with an average diameter of 30.5 ± 0.2 nm (Figure S1A), together with a red shift of the surface plasmon resonance peak in UV-Vis spectrum (Figure S1B), infers successful functionalization of AuNPs with the oligo [1,2].

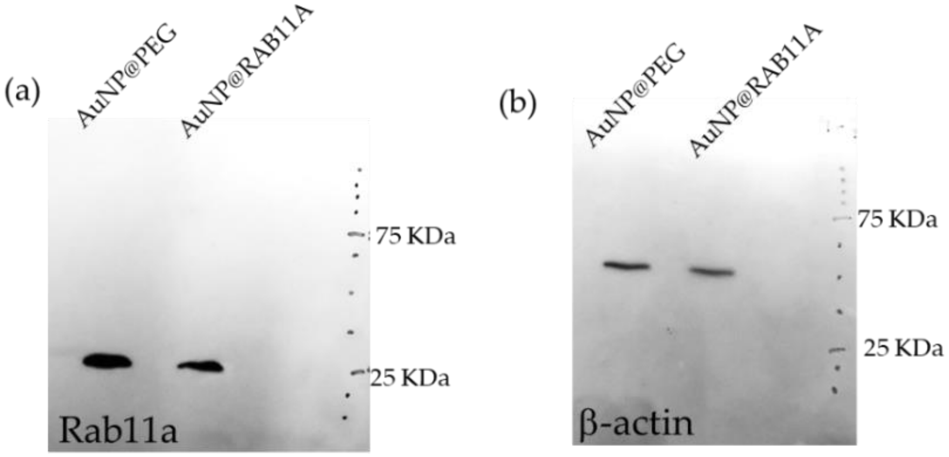

Figure S2. Western-blot for quantification of RAB11a protein. Western blot analysis of the expression of RAB11a and β-actin in K562 cell line after 12h incubation with gold nanoparticles covered with PEG (AuNP@PEG) and functionalized with anti-*RAB11A* (AuNP@RAB11A). (**a**) Western blot of Rab11a protein with 25 KDa; (**b**) Western blot of β-actin protein with 46 KDa.

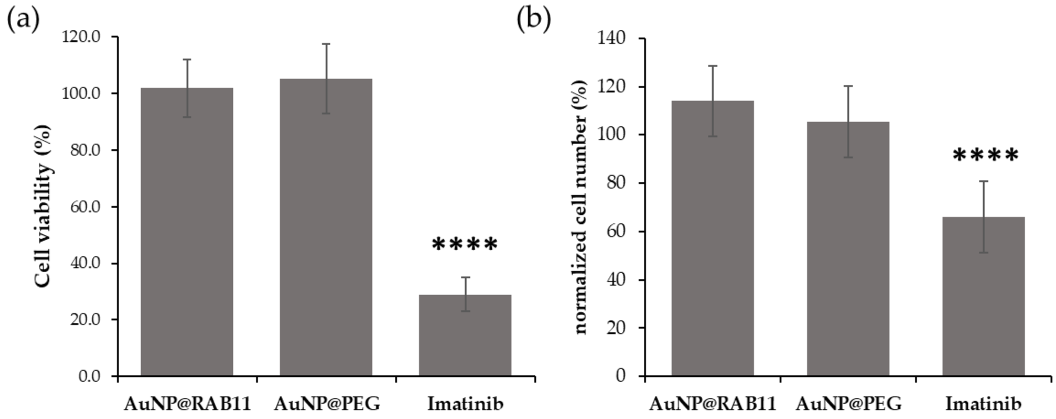

Figure S3. K562 cell viability. The cell viability was measured after 24h incubation with gold nanoparticles covered with PEG (AuNP@PEG) and functionalized with anti-*RAB11A* (AuNP@RAB11A). **(a)** Cell viability using the MTS colorimetric assay. **(b)** percentage of cells measured using the Trypan blue exclusion method. Data was normalized to the respective control - untreated cells was the control of nanoformulations treated cells, and cells treated with 0.1% (v/v) DMSO was the control of imatinib treated cells. **** p-value < 0.0001 relative to the respective control.

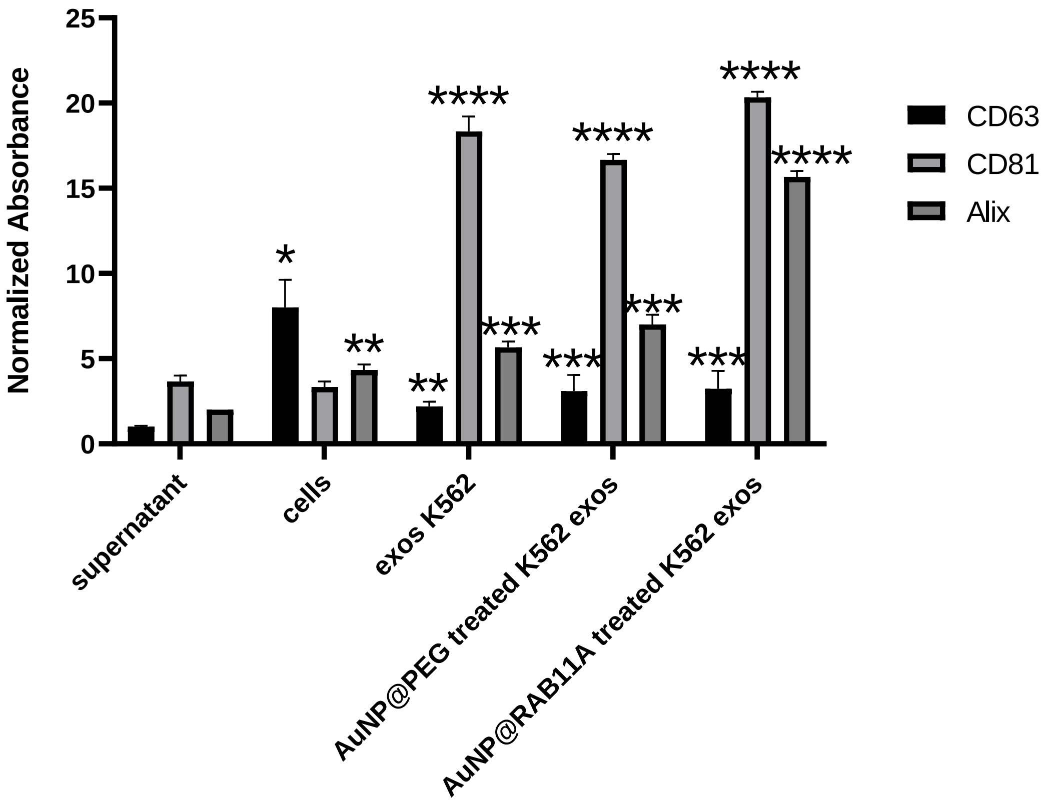

Figure S4. Quantification of CD63, CD81 and Alix proteins in exosomes suspensions. Normalized absorbance of ELISA analysis to infer the presence of CD63 (black bars), CD-81 (light grey bars) and Alix (dark grey bars) in exosomes of K562 cells incubated for 12h with 0.45 nM AuNP@PEG or AuNP@RAB11A, or untreated (exos K562). The supernatant of untreated K562 cells previous to exosome extraction, K562 cell extract and medium (exo-DMEM) were also analyzed for control purposes. Bars represent the average ± standard deviation of Absorbance 450 nm normalized to values obtained for medium. * p-value < 0.05, ** p-value < 0.01, *** p-value < 0.001, **** p-value < 0.0001 relative to respective supernatant.

The increased normalized absorbance in exosome samples, suggests the increased abundance of the CD63, CD81 and Alix proteins, and hence the successful exosome extraction, according to MISEV18 guidelines [3].

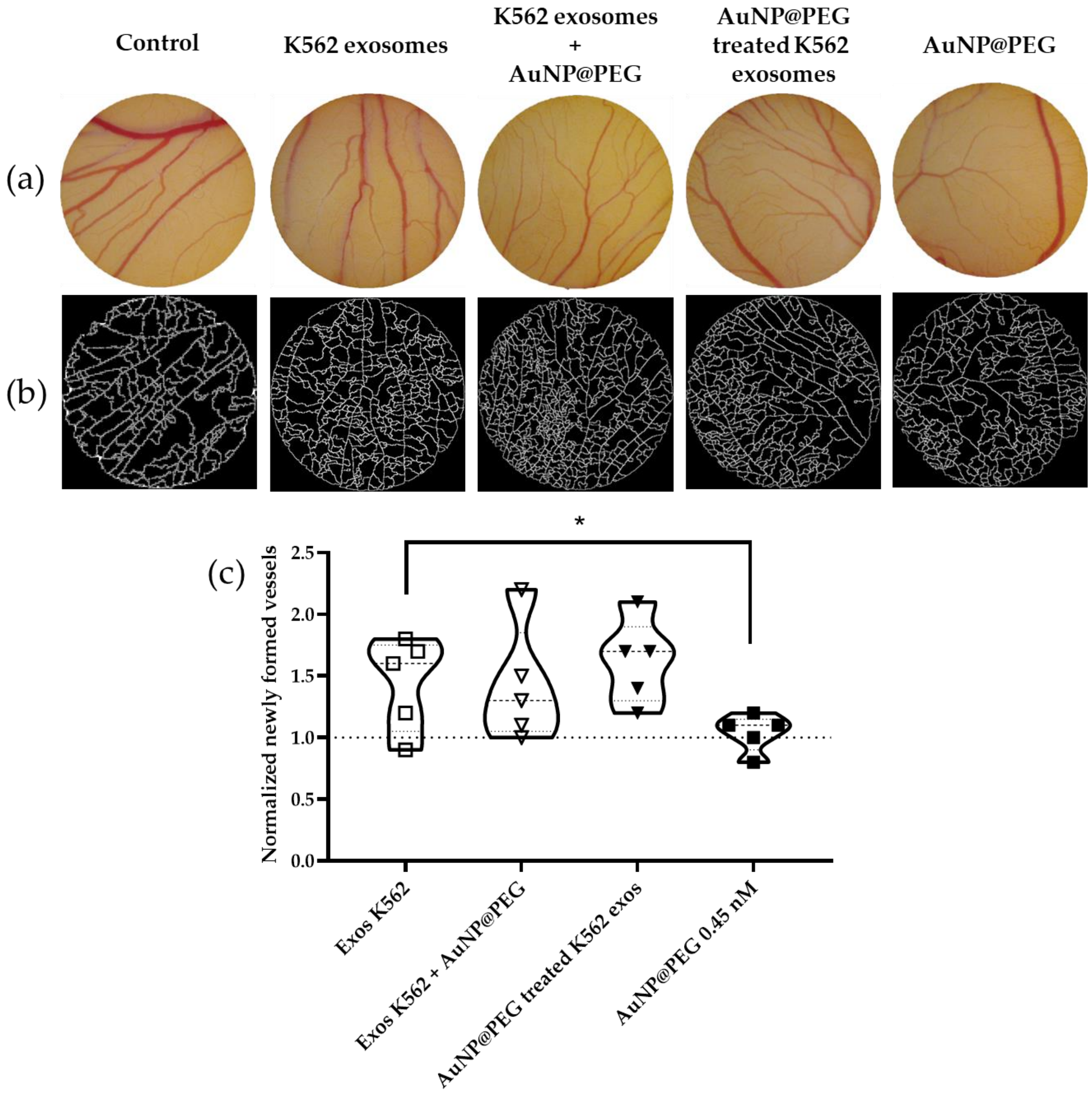

Figure S5. Angiogenic potential of K562 exosomes (controls). Evaluation of angiogenic potential of K562 exosomes ($1x10^9$ exosomes; Exos K562), a mixture of K562 exosomes ($1x10^9$ exosomes) with 0.45 nM AuNP@PEG (Exos K562 + AuNP@PEG), exosomes collected from K562 exposed for 12h to 0.45 nM AuNP@PEG (AuNP@PEG treated K562 exos), or to 0.45 nM AuNP@PEG. (**a**) Aspect of the chorioallantoic membrane (CAM) exposed for 24h with with control (Phosphate buffer saline, PBS), Exos K562, Exos K562 + AuNP@PEG, AuNP@PEG treated K562 exos or AuNP@PEG with 4x magnification. (**b**) Segmented image of the respective CAM region region used to calculate number of branches; (**c**) Violin density plots of newly formed vessels in CAMs exposed for 24h to Exos K562 (squares), Exos K562 + AuNP@PEG (white triangles), AuNP@PEG treated K562 exos (dark triangles) or AuNP@PEG (dark circles), normalized to CAM regions incubated with vector control (PBS) and to the respective CAM at 0h timepoint. Dotted line at 1.0 normalized newly formed vessels refers to control sample (region of the CAM incubated with PBS after 24h normalized to respective CAM at 0h). * p-value < 0.05.

References:

1. Conde, J.; Rosa, G.; Baptista, P.V. Gold-Nanobeacons as a Theranostic System for the Detection and Inhibition of Specific Genes. *Nat. Protoc. Exch.* 2013. Available online: https://www.nature.com/protocolexchange/protocols/2881 (accessed on 1 February 2022).
2. Conde, J.; Rosa, J.; de la Fuente, J.M.; Baptista, P.V. Gold-nanobeacons for simultaneous gene specific silencing and intracellular tracking of the silencing events. *Biomaterials* **2013**, *34*, 2516–2523. https://doi.org/10.1016/j.biomaterials.2012.12.015.
3. Clotilde Théry, C.; Witwer, K.W.; Aikawa, E.; Alcaraz, M.J.; Anderson, J.D.; Andriantsitohaina, R.; Antoniou, A.; Arab, T.; Archer, F.; Atkin-Smith, G.K.; et al. Minimal information for studies of extracellular vesicles 2018 (MISEV2018): a position statement of the International Society for Extracellular Vesicles and update of the MISEV2014 guidelines. *J. Extracell. Vesicles* **2018**, *7*, 1535750. https://doi.org/10.1080/20013078.2018.1535750.
